# Outcomes of retained and disengaged pregnant women living with HIV in Uganda

**DOI:** 10.1371/journal.pone.0251413

**Published:** 2021-05-21

**Authors:** Agnes N. Kiragga, Ellon Twinomuhwezi, Grace Banturaki, Marion Achieng, Juliet Nampala, Irene Bagaya, Joanita Kigozi, Barbara Castelnuovo, Beverly S. Musick, Rohan Hazra, Constantin T. Yiannoutsos, Kara K. Wools-Kaloustian

**Affiliations:** 1 Research Department, Infectious Diseases Institute, College of Health Sciences, Makerere University, Kampala, Uganda; 2 School of Medicine, Indiana University, Indianapolis, Indiana, United States of America; 3 National Institute of Child Health and Human Development (NICHD), National Institutes for Health, Bethesda, MD, United States of America; 4 Fairbanks School of Public Health, Indiana University, Indianapolis, Indiana, United States of America; 1. IRCCS Neuromed 2. Doctors with Africa CUAMM, ITALY

## Abstract

**Introduction:**

Loss-to-follow-up among women living with HIV (WLWHIV) may lead to unfavorable outcomes for both mother and exposed infant. This study traced WLWHIV disengaged from care and their infants and compared their outcomes with those retained in care.

**Methods:**

The study included WLWHIV who initiated ART during pregnancy at six public clinics in Uganda. A woman was defined as disengaged (DW) if she had not attended her 6-week post-partum visit by 10 weeks after her estimated date of delivery. DW were matched with retained women (RW) by age and duration on ART. Nurse counselors traced all selected DW via telephone and community visits to assess vital status, infant HIV sero-status and maternal HIV viral load through blood draws.

**Results:**

Between July 2017 and July 2018, 734 women (359 DW and 375 RW) were identified for the study. Tracing was attempted on 349 DW and 160 (44.6%) were successfully located and enrolled in the study. They were matched with 162 RW. Among DW, 52 (32.5%) transferred to another health facility. Very few DW, 39.0% were HIV virally suppressed (<1000 copies/ml) compared to RW 89.5%, P<0.001). Among 138 babies born to DW, 4.3% tested positive for HIV compared to 1.4% among babies born to RW (P = 0.163).

**Conclusion:**

Pregnant and breastfeeding WLWHIV who disengage from care are difficult to find in urban environments. Many have detectable viral loads, leading to the potential for an increased risk of MTCT. Efforts to reduce disengagement from care are critical for the successful elimination of MTCT in resource-limited settings.

## Introduction

Provision of lifelong antiretroviral therapy (ART) to all pregnant women living with HIV, initially under the Option B+ policy and now under Treat All, has led to a 48% reduction in mother-to-child transmission (MTCT) in sub-Saharan Africa (SSA) [1,2], and has averted 1.4 million new HIV infections since 2010 [2]. In 2019, data from UNAIDS showed that 93% of pregnant women living with HIV in Eastern and Southern Africa received ART, resulting in low MTCT rates [3]. Despite these successes, in 2018, 180,000 children became infected with HIV globally [3], undermining the target of an HIV-free generation. MTCT is often the result of mothers disengaging from care following ART initiation [4–7], with infections occurring during the breastfeeding and postnatal periods rather than during pregnancy [8]. In 2019, 38 000 new infections occurred in UNAIDS focus African countries because women living with HIV did not receive antiretroviral therapy during pregnancy and 29 000 because women dropped out of antiretroviral therapy [9]. Disengagement from care may lead to unfavorable outcomes and true transmission rates are likely to be underestimated if women and infants who are lost to follow-up (LTFU) are not accounted for. HIV programs may underestimate transmission as women who are not in care may not be on ART, not virally suppressed, with higher risks of MTCT.

Several evaluations of country-specific prevention of mother-to-child transmission (PMTCT) programs have been performed in SSA, in countries such as Malawi [10,11], Nigeria and South Africa [12]. In Kenya, the PMTCT program led to a decline in maternal HIV transmission from 10.9% to 2.5% [13]. In Uganda, the percentage of children born to women living with HIV (WLWHIV) who become infected is estimated at 2.5% at 6 weeks post-partum and 5.3% at the end of the breastfeeding period [14]. Nevertheless, while most countries have achieved reductions in the rate of vertical HIV transmission, they have also reported high attrition among women initiating ART during pregnancy [15].

Studies conducted in Kenya and South Africa found that 76% of women adhered to ART during pregnancy, but only 53% returned for post-partum clinic appointments [16]. In Malawi, women initiating ART within PMTCT programs were five times more likely to drop out of care compared to non-pregnant women [17]. However, little is known about maternal and infant outcomes once a woman is LTFU. Understanding the impact of disengagement during pregnancy and the early post-partum period on maternal and infant outcomes is critical to assessing the efficacy of initiatives like Option B+ and Treat All, and for generating more accurate global estimates of MTCT of HIV.

Several tracing studies have addressed the outcomes of non-pregnant adults who have become LTFU [18–21]. However, other than three studies from Zimbabwe, eSwatini and Malawi, there is a dearth of knowledge about the outcomes of pregnant women initiating ART who subsequently become LTFU [22–24]. Such outcomes may include estimates of HIV viral suppression, MTCT and care status among women who disengage from HIV care programs. Given the paucity of data, this study aimed to trace women who disengaged from PMTCT services through community outreach. Our goals were to compare maternal and infant outcomes, such as vertical transmission and maternal HIV viral suppression, between women who were disengaged and those who were retained in care, and to identify the reasons for disengagement.

## Methods

### Ethical considerations

The study received approval from the Joint Clinical Research Center Institutional Review and Ethics Committee, the Uganda National Council of Science and Technology (Ref: HS35ES) and the Indiana University Institutional Review Board. All enrolled women provided written informed consent for study participation for themselves and their infants.

### Study site and population

The study was conducted in Uganda at six public Kampala City Council Authority (KCCA) clinics supported by the Infectious Diseases Institute (IDI) and the Uganda Centers for Disease Control-President’s Emergency Plan for AIDS Relief (CDC-PEPFAR) HIV care program. Study activities leveraged the routine services offered at the PMTCT clinics for newly diagnosed pregnant WLWHIV, which included pre-ART adherence counseling, and same-day ART initiation *of tenofovir+lamivudine+efavirenz* (TDF+3TC+EFV), with a two-week return appointment. HIV viral load testing is done 6 months after ART initiation and yearly thereafter, as per Uganda National HIV Viral load testing algorithm. Women undertake monthly follow-up appointments during pregnancy and throughout the first 18 months of the post-partum period, consistent with the national Maternal Child Health (MCH) schedule for both women and HIV exposed infants [25,26]. WLWHIV 18 years of age or older, who initiated ART during pregnancy and were at least 6–12 weeks post-partum but less than 36 weeks were eligible for the study. The gestational age was estimated based on the date of the woman’s first antenatal clinic and database closure. Women known to have died or transferred out to another HIV care facility were excluded.

### Study design

This was a prospective cohort study that enrolled women retained and disengaged from HIV care. A woman was considered disengaged (DW) if she had not had a clinic visit in the 90 days preceding database closure and had not returned for her scheduled 6-week postpartum visit by 10 weeks post-partum. The time point of the disengagement was assigned at 90 days after the woman’s last scheduled clinic appointment. The DW were further categorized into two groups; women who delivered while in care and those who disengaged before delivery. The estimated date of delivery (EDD) was used to determine the postpartum time frame for women who disengaged before delivery.

A woman was considered retained (RW) if she was actively attending the health care facility with at least one clinic encounter within 90 days of database closure and had attended her 6-week post-partum visit. All disengaged women were matched one-to-one with a retained woman by age (plus or minus 5 years) and months from ART initiation to study enrollment (plus or minus one month). Two retained women were erroneously included in the study prior to community tracing and enrollment of their matched disengaged women, causing an excess of RW. Age and duration on ART were selected for matching, as these are known to be associated with for disengagement from PMTCT programs [27]. Potential facility level factors associated with disengagement from care were well balanced since all the study sites serve similar catchement areas around Kampala city, and have similar governance and staffing structures.

### Study procedures

Using information recorded in the Uganda Electronic Medical Record (UgandaEMR) [28] available at each of the study sites, all women who met the definition of DW or RW were flagged. All DW were first contacted through a telephone call to confirm personal details prior to community tracing and to ascertain their willingness to be traced. If uncontactable over the telephone, the study nurse counselor and one member from the health facility outreach team proceeded to the community using information recorded in the mother’s clinic file. During community outreach, confidentiality of a mother’s HIV status was maintained, and the study team identified themselves as representatives from the national MCH program. Blood samples were collected from the disengaged women from home or at the health facility, and their infants for HIV viral load and HIV DNA PCR testing, respectively. A viral load of <1000 copies/ml was considered undetectable. All DW who reported not being in care were encouraged to re-engage in care with their infant and were re-contacted one month after study enrollment to determine if they linked back to care and results of their laboratory testing provided to them during their next clinic appointment or via the telephone or a home visit if preferred.

For DW who reported that they had transferred to another HIV care and treatment facilities, part of the collected blood samples were used to determine drug levels of efavirenz using high performance liquid chromatography with ultra-violent detection. Drug levels were performed among disengaged women who reported to have transferred care, as way of validating their care status with the amount of drug levels. Retained women whose age and duration from ART initiation matched those of the DW who reported to have transferred to another facility were similarly tested for efavirenz drug levels.

Retained women were contacted via telephone, approached by the nurse counselor during their next clinic appointment and enrolled. Similarly, blood samples were collected from RW and their infants for HIV viral load and HIV DNA PCR testing, respectively. All infants were enrolled in the study if they were less than 9 months old. Results from the laboratory investigations were provided to the retained women approximately two weeks after study enrolment.

### Data collection

The REDCap mobile application using Android-powered computer tablets was used for study data collection and storage. Information was collected using the UgandaEMR [28], and structured questionnaires. Information extracted from the UgandaEMR included dates of clinic registration and ART initiation plus clinical variables (e.g. weight, CD4 cell count, WHO clinical stage) at ART initiation and subsequent clinic encounters. The structured questionnaires collected information on infant HIV testing, breastfeeding and complimentary feeding practices, experience of intimate partner violence and disclosure of HIV status. Additional information on engagement in care status at the time of outreach and reasons for disengagement from care were collected from the DW.

### Statistical analysis

We compared demographic and clinical characteristics between DW and RW at ART initiation and at study enrollment. We used chi-square tests for associations between two categorical variables and the Fisher’s Exact for small cell sizes (expected values less than 5). The Kruskal Wallis test was used to compare differences in continuous and ordinal variables between the two groups. All analyses were performed using the STATA software version 15.2 (Stata Corp, College Station, TX).

## Results

### Outcomes of retained and disengaged women

**Fig 1:**
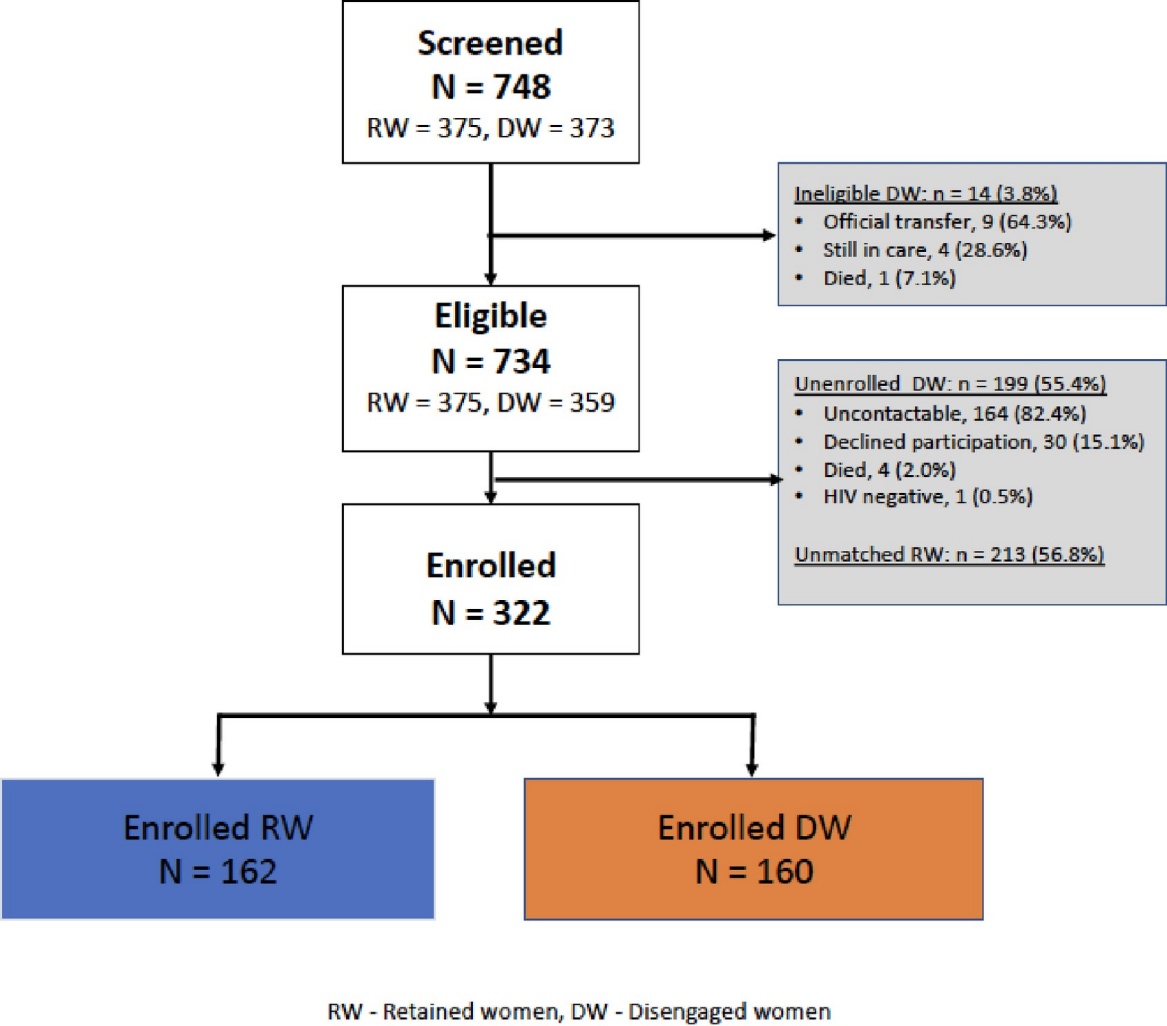
Study cohort diagram showing HIV positive retained and disengaged women enrolled in a community outreach study between July 2017 –July 2018 from six Kampala City Council public municipal clinics in Uganda.

Between July 2017 and July 2018, a total of 748 WLWHIV were initiated on ART during pregnancy and enrolled in the PMTCT program at all the six KCCA clinics in Kampala, with 375 ultimately being retained and 373 being disengaged. Of these, 734 (375 RW and 359 DW) were eligible for the study (**Fig 1**). Among eligible RW, 213 (56.8%) were not matched with a DW and were not enrolled in the study. Of 199 DW (55.4%) who were not enrolled, 164 (82.4%) were uncontactable, 30 (15.1%) declined participation, 4 (2.0%) were found to be deceased and 1 (0.5%) woman subsequently tested HIV negative. Consequently, a total of 322 women (162 RW and 160 DW) were enrolled in the study (**Fig 1**). Retained and disengaged women had similar age, marital status and CD4 count at ART initiation, while RW had a slightly longer duration from ART initiation at study enrolment, median 9.5 months (IQR: 8.1,10.9) compared to 9.0 months (7.7, 10.5) among DW, P = 0.026 (**Table 1**). A larger proportion, 58.1%, of DW had a low education level (primary or none) compared to RW 37.7%, P<0.001, and 152 (95.0%) DW initiated ART later in their pregnancy (in the second or third trimester) as compared to 139 RW (85.8%; P = 0.005).

**Table 1:** Demographic and clinical characteristics of retained and disengaged women at study enrollment.

Characteristic	Retained Women (RW) (N = 162)	Disengaged Women (DW) (N = 160)	P Value
Age in years, Median (IQR)	26.3 (23.6–29.1)	25.6 (22.8–28.4)	0.123
Marital status^1^, n (%)			
Single	12 (9.8)	8 (7.3)	0.123
Married/cohabiting	87 (71.3)	68 (62.4)	
Separated/divorced/widowed	23 (18.9)	33 (30.3)	
Education level, n (%)			
None/primary	61 (37.7)	93 (58.1)	<0.001
Secondary	91 (56.2)	61(38.1)	
Tertiary	10 (6.2)	6 (3.8)	
CD4 cells/uL at ART start^2^, Median (IQR)	445 (319, 600)	501 (324, 636)	0.442
Trimester at ART start, n (%)			
I	23 (14.2)	8 (5.0)	0.005
II/III	139 (85.8)	152 (95.0)	
Duration in months between ART initiation and study enrollment, Median (IQR)	9.5 (8.1,10.9)	9.0 (7.7–10.5)	0.026

Note

^1^Marital status collected among 122 RW and 109 DW.

^2^CD4 cell counts at ART initiation available for 119 RW and 79 DW, IQR interquartile range, ART Antiretroviral therapy.

Among the 160 DW enrolled in the study, 52 (32.5%) reported that they were receiving care at another health facility (self-transfers) (**Table 2**). The majority of DW (99; 71.7%) delivered after they had disengaged from care, while 39 (28.3%) delivered while in care (**Table 2**). Both groups of DW were similar with regard to age, education level, and marital status. Upon tracing, 24 (61.5%) women who delivered before disengagement and 69 (69.7%) who delivered after disengagement, had still not re-engaged in care anywhere else. Among the 22 DW who did not have babies enrolled, 11 (50.0%) delivered their infant or experienced the respective pregnancy outcome before disengaging from care.

**Table 2:** Maternal outcomes among retained and disengaged women in a PMTCT program in Uganda.

Maternal Outcomes	Retained Women (RW) (N = 162)	Disengaged Women (DW) (N = 160)	P value
Timing of disengagement			
Delivered before disengaged, n (%)	N/A	39 (28.3)^**1**^	N/A
HIV Viral load			
VL < 1000 copies/ml, n (%)	145 (89.5)	62 (39.0)^**2**^	<0.001
Care status at successful outreach, n (%)			
In care elsewhere (Transfers)	N/A	52 (32.5)	N/A
Detectable Efavirenz concentrations^**3**^, n (%)	45 (90.0)	36 (72.0)	0.022
Post-partum family planning use, n (%)	57 (35.2)	36 (22.5)	0.012
Disclosed HIV status to anyone, n (%)	109 (67.3)	87 (54.4)	0.018
Disclosed HIV status to spouse^4^, n (%)	75 (68.8)	48 (55.2)	0.050

^1^Out of 138 women with data on timing of delivery.

^**2**^One DW had missing HIV viral load result.

^3^Out of 50 DW reporting being in care and 50 matched RW.

^4^Among the 109 RW and 87 DW who had disclosed their status.

### HIV viral suppression

Of women with viral load results (DW 159, RW 162), DW had significantly lower (39.0%) HIV viral suppression rates compared to RW (89.5%; P<0.001 (**Table 2**). Of the 52 DW who had self-transferred, 51 had HIV viral load results and a greater percentage of these, 70.6% (36/51), had achieved viral suppression compared to 24.1% (26/108) of those who were out of care (P<0.001).

Fifty of the DW who self-transferred and their matched RW were tested for detectable drug levels. Of the 50 DW, 36 (72.0%) had detectable efavirenz drug concentration. Among the 50 matched RW, 45 (90.0%) had detectable efavirenz drug concentrations, P = 0.022 (**Table 2**). HIV viral suppression was higher 78/81 (96.3%) among women with detectable efavirenz levels compared to 2/19 (10.5%) of women with no efavirenz concentrations, P<0.001.

### HIV status disclosure and uptake of post-partum family planning

Among the 160 DW, 48 (55.2%) had disclosed their HIV status to their partner, compared to 75 of 162 RW (68.8%; P = 0.050) (**Table 2**). Among DW, 36 women (22.5%) were using any family planning method during the postpartum period compared to 57 RW (35.2%; P = 0.012) (**Table 2**).

### Follow-up of disengaged women

An attempt was made to contact all disengaged women one month after study enrollment; at that time 10 (6.3%) were uncontactable. Despite initial contact and extensive counseling, 37 (24.7%) of the remaining 150 DW had not re-engaged in care. Reasons for not re-engaging were not mutually exclusive and included lack of transport to the health facility (41.6%), fear of pill burden (33.3%), denial of HIV status (12.8%), belief in spiritual healing (7.7%), HIV negative status of infant (2.6%) and use of herbal medicine to treat HIV (2.6%).

### Outcomes of babies born to retained and disengaged women

**Fig 2:**
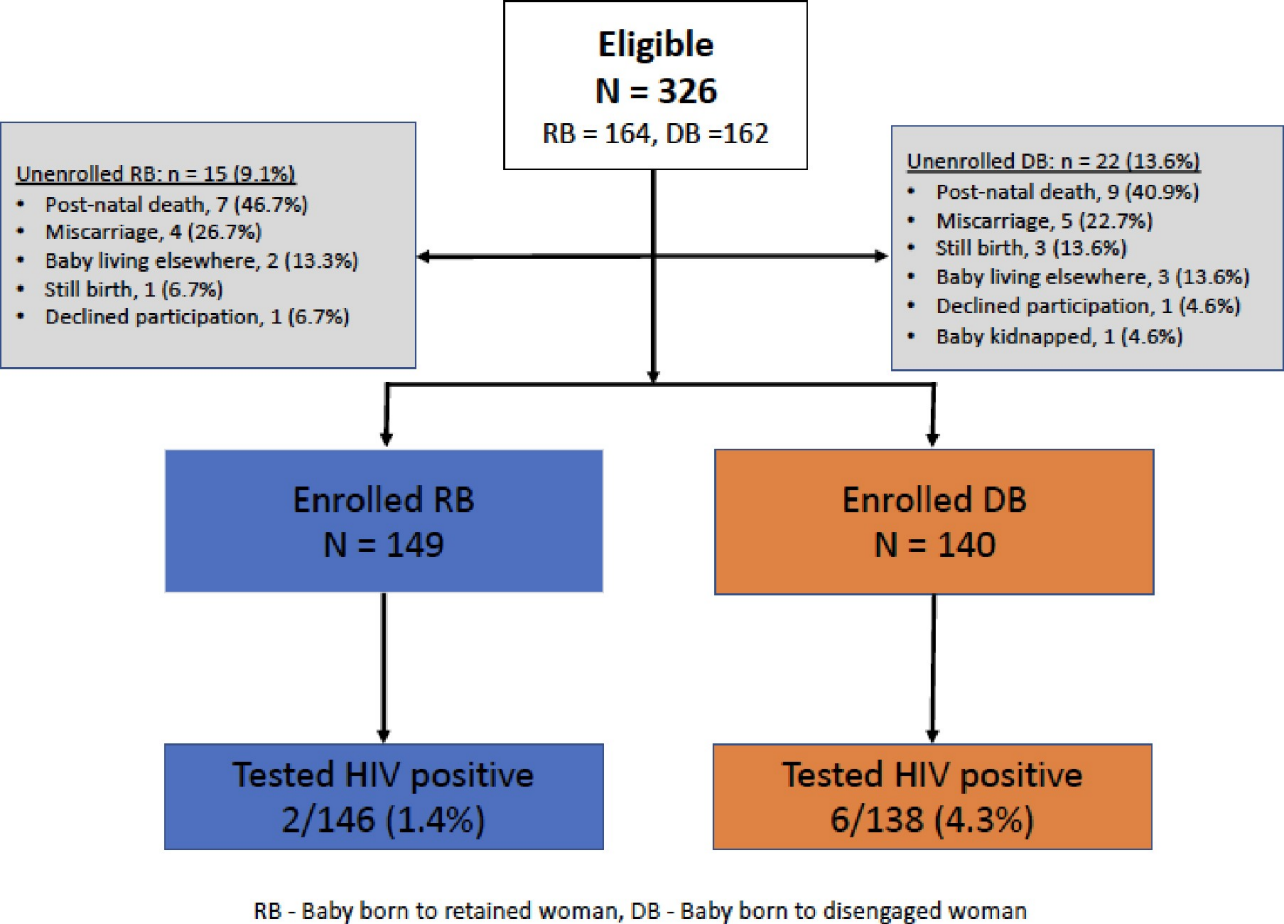
Study consort diagram showing infants born to HIV positive retained women (RB) and disengaged women (DB) enrolled in a community outreach study between July 2017 –July 2018 from six Kampala City Council public municipal clinics in Uganda.

A total of 326 fetuses (164 RW and 162 DW) were observed among the 322 enrolled women, four pregnancies (2 DW and 2 RW) were twins. Among the 162 pregnancies in RW, 149 (90.9%) babies were alive and enrolled, 3 (1.8%) were not enrolled, while 12 (7.3%) pregnancies had adverse outcomes (7 post-natal deaths, 1 still birth and 4 miscarriages). Among the 3 infants who were not enrolled, 2 were not living with their mothers and one was declined enrollment by the mother. Among the 160 pregnancies in DW, 140 (86.4%) babies were alive and enrolled, 5 (3.1%) were not enrolled, and for 17 (10.5%) pregnancies had adverse outcomes (9 post-natal deaths, 3 still births and 5 miscarriages). Among the 5 infants of DW who were not enrolled, 3 were not living with their mothers, 1 was declined enrollment and 1 baby was missing/kidnapped (**Fig 2**). None of the babies was found to be more than 9 moths (36 weeks) of age. There were no statistically significant differences in the occurrence of adverse pregnancy outcomes between RW and DW (P = 0.314).

Infants born to retained and disengaged women were similar in gender distribution and age at enrollment with a median of 6 months and similar proportions in both groups were exclusively breast feeding at the time of study enrollment. A significantly lower proportion of babies of DW (38; 27.1%) were enrolled in the early infant diagnosis programs compared to 144 of the babies born to RW (96.6%; P<0.001), (**Table 3**). Among 138 of the babies of DW with an HIV test, 6 (4.3%) tested positive for HIV compared to 2 among 146 (1.4%) babies of RW (P = 0.162) (**Table 3**). Among 39 DW who delivered prior to disengagement from care one baby was found to be HIV-positive (MTCT rate 2.6%), compared to 5 babies (MTCT rate 5.1%) among women who delivered after disengagement.

**Table 3:** Demographic, clinical characteristics and outcomes of babies born to retained and disengaged women in a PMTCT program in Uganda.

Characteristic	Babies of Retained Women (N = 149)	Babies of Disengaged Women (N = 140)	P Value
Age (months) at study enrollment, Median (IQR)	6.0 (4.4, 7.5)	5.4 (4.4, 6.7)	0.282
Male sex, n (%)	84 (56.4)	74 (52.9)	0.548
Weight in Kg at birth, Median (IQR)	3.2 (3.0, 3.5)	3.0 (2.9, 3.5)	0.259
Place of birth, n (%)			
Health facility	145 (97.3)	130 (92.9)	0.205
Home	3 (2.0)	8 (5.7)	
Not Documented	1 (0.7)	2 (1.4)	
Infant feeding at study enrollment, n (%)			
Exclusively breastfeeding	68 (45.6)	56 (40.0)	0.334
Enrolled in Early Infant Diagnosis care^**1**^, n (%)	144 (97.9)	38 (27.5)	<0.001
**Infant outcomes**			
Median weeks at PCR testing	24 (19, 35)	22 (21, 34)	0.146
HIV PCR Positive^**2**^, n (%)	2 (1.4)	6 (4.3)	0.163
HIV PCR positivity by timing of delivery, n (%)	N/A		N/A
Delivered before mother disengaged		1/39 (2.6)	
Delivered after mother disengaged		5/99 (5.1)	

^**1**^Out of 147 retained babies and 138 disengaged babies.

^**2**^Unusable samples for HIV PCR test for 3 retained and 2 disengaged babies.

## Discussion

In our study, we observed that nearly half of women engaged in PMTCT programs had disengaged from care. These estimates were comparable to countrywide evaluation of the Option B+ program which reported similar loss-to-follow-up among pregnant women initiating ART in Uganda [29]. Despite the intensive outreach of disengaged pregnant women enrolled in the PMTCT program, we were only able to ascertain outcomes of 44.6% of these women. Tracing studies in non-pregnant adults have reported higher rates of successful tracing, several with over 80% outcome ascertainment for the outreached population [21,30,31]. In eSwatini, community tracing of women who were LTFU ascertained outcomes in 44.7% of women [23], while in Malawi only 40% of women LTFU in Option B+ programs were successfully traced [24]. These low rates of outcome ascertainment highlight the challenges of community tracing of WLWHIV in SSA.

One of the most common reasons for failure to ascertain outcomes of the pregnant women may be their high mobility. High mobility and migration of persons living in urban communities is attributed to the search for employment, particularly among young adults [32,33]. Among pregnant women, nearly half return to their original home for delivery and family support during the postpartum period [34]. This contributes to the difficultly in locating DW. In a study in South Africa, the majority of women traveled away from the city to the villages of origin for a relatively short time (median of 32 days), and planned to return to their original health facility in the city, while infant care was often delegated to the grandparents [34]. Understanding the mobility patterns of postpartum women thus remains critical for ensuring continuity of care for WLWHIV and their exposed infants. Additionally, it is important to identify and address resources and relational health system barriers to retention in care among WLWHIV [35,36].

As expected, we observed that a higher (61.0%) proportion of DW had detectable VL as compared to RW (10.4%). This was the case even among women who reported receiving care at another facility, of whom 28.4% had unsuppressed virus compared to 9.7% for matched RW. In a recent study in Zambia, viremia was present in 18.1% of people living with HIV/AIDS (PLWHV) who were retained in care, 71.3% in those LTFU, 49.8% of those lost and in care elsewhere and 83.9% of those lost and not in care [37]. Such high levels of unsuppressed viremia among DW increases the risk of HIV vertical transmission [38]. We postulate the rates of viral non-suppression for untraceable women are as high or higher than those who were successfully traced. This is assumption is based on the results from Sikazwe et al, where they found that 71.3% of persons who were lost had high viremia compared to those retained [34]. The low ascertainment of outcomes among WLWHIV who disengage from care underscores the need for strategies that identify women who are likely to disengage from care soon after ART initiation and that ensure retention, particularly during the postpartum period.

In our study, we found that 67.5% of the DW were out of care and had not received any ART since their last clinic encounter, while 32.5% had self-transferred to other health facilities. Our estimate of self-transfer was higher than that reported in a systematic review of data from 23 low-income and middle-income countries where 18.6% of the persons LTFU had silently transferred [39]. Our data cannot shed light as to whether this difference in our experience is a fact inherent to patterns of care seeking behavior in our setting or simply a positive bias in our estimates due to the large number of women whom we were unable to trace (if these women have lower probability of accessing care after dropping out from their original healthcare facility). Nevertheless, we found that 28.4% of the women who reported being in care elsewhere had unsuppressed viremia. PLWHIV who self-transfer face challenges of continuing ART at new health facilities and this is likely to affect adherence to treatment [34,37]. In fact, in our study, among women who reported that they had transferred, nearly a third had undetectable ART drug concentrations compared to 10% among women who were retained in care (P = 0.022). This discordance may represent issues related to women falsely reporting that they are in care based on social desirability. Studies have shown large discordances between ART detection and self-report adherence, and such discrepancies are more common among young adults and the results may be subject to social desirability bias [40,41]. Health care workers should emphasize the importance of continuity in care among WLWHIV. WLWHIV should be encouraged to seek ART services at facilities that are convenient to them in order to and avoid complete disengagement from care.

Although our study did not have adequate power to detect differences in vertical transmission of HIV, our findings suggest that higher transmission in infants born to DW as compared to RW. Our estimates of HIV transmission among DW were higher than that reported in a study conducted among women who disengaged from care in Zimbabwe, where the observed estimates of 3.6% were reduced to 1.8% following a tracing program which used community workers to ensure that all HIV positive pregnant women were in care throughout the post-partum period [22]. Among the women retained in care, our estimates of vertical transmission are slightly lower than Uganda national estimates of 2.9% [42]. In the Southern Africa, comparable rates of infant HIV infection (3%) were observed at 12 months and HIV-free survival was estimated at 97.5% at 6 months [43]. Even with the scarcity of prior studies reporting HIV vertical transmission among DW, our findings are likely optimistic as there are concerns that women that couldn’t be traced might have higher transmission rates for reasons described earlier. These results highlight the value of outreach activities for pregnant and breastfeeding WLWHIV.

## Study strengths and limitations

One of the strengths of our study is that it contributes to our understanding of HIV outcomes among pregnant and breastfeeding women who disengage from HIV programs in SSA. Our findings are generalizable to populations in urban areas in SSA with great population mobility. We also acknowledge some limitations of our study. From the electronic health records, almost half of women who were identified as disengaged from care were not successfully contacted, leading to a much smaller sample size than was initially planned. Because of this, we were underpowered to fully explore questions related to MTCT risk factors and drug concentrations. Also, the differences in the months of postpartum led to different amounts of person time which could be have contributed to the non-statistical difference in MTCT. More importantly, some of our findings may be biased because of a strong association between an outcome (e.g. re-engagement in care) and whether a woman was successfully traced. In addition, our study cannot address outcomes in less mobile, non-urban settings.

## Conclusions

High rates of disengagement from care were observed among women enrolled in PMTCT programs in Uganda. Pregnant and breastfeeding women who disengage from care are difficult to find in urban environments and a large proportion of the traceable women are out of care and have detectable viral loads leading to the potential for an increased risk of MTCT. In the current era of “Treat All”, development of strategies that enhance retention, mechanisms that track silent and unofficial transfers, as well as estimation of viral suppression among persons who disengage from care are critical. Customized interventions are needed to target pregnant women initiating ART that address several psychosocial and structural barriers to retention, particularly in the post-partum period. These results underscore the need for community tracing of women living with HIV following disengagement from care and highlight the poor outcomes associated with dropout for both mothers and their infants. Our study findings were used to inform a national campaign titled “Free to Shine Campaign” which which aimed to keep mothers and babies in care, and to prevent new HIV infections [44]. Similar campaigns coupled with tracing efforts will be critical for successful elimination of MTCT in resource-limited settings.

## Supporting information

S1 Dataset (CSV)
